# Nanocrystalline Antiferromagnetic High-κ Dielectric Sr_2_NiMO_6_ (M = Te, W) with Double Perovskite Structure Type

**DOI:** 10.3390/molecules25173996

**Published:** 2020-09-02

**Authors:** Jelena Bijelić, Dalibor Tatar, Sugato Hajra, Manisha Sahu, Sang Jae Kim, Zvonko Jagličić, Igor Djerdj

**Affiliations:** 1 Department of Chemistry, Josip Juraj Strossmayer Univesity of Osijek, Cara Hadrijana 8/A, HR-31000 Osijek, Croatia; jelena.bijelic@kemija.unios.hr (J.B.); tatar.dalibor42@gmail.com (D.T.); 2 Nanomaterials and System Lab, Major of Mechatronics Engineering, Faculty of Applied Energy Systems, Jeju National University, Jeju 63243, Korea; sugatofl@outlook.com (S.H.); manishafl@outlook.com (M.S.); kimsangj@jejunu.ac.kr (S.J.K.); 3 Institute of Mathematics, Physics and Mechanics, University of Ljubljana, Jadranska 19, SI-1000 Ljubljana, Slovenia; zvonko.jaglicic@imfm.si; 4 Faculty of Civil and Geodetic Engineering, University of Ljubljana, Jamova 2, SI-1000 Ljubljana, Slovenia

**Keywords:** antiferromagnet, double perovskite, high-κ dielectric, nickel, tellurium, tungsten

## Abstract

Double perovskites have been extensively studied in materials chemistry due to their excellent properties and novel features attributed to the coexistence of ferro/ferri/antiferro-magnetic ground state and semiconductor band gap within the same material. Double perovskites with Sr_2_NiMO_6_ (M = Te, W) structure type have been synthesized using simple, non-toxic and costless aqueous citrate sol-gel route. The reaction yielded phase-pure nanocrystalline powders of two compounds: Sr_2_NiWO_6_ (SNWO) and Sr_2_NiTeO_6_ (SNTO). According to the Rietveld refinement of powder X-ray diffraction data at room temperature, Sr_2_NiWO_6_ is tetragonal (*I*4/*m*) and Sr_2_NiTeO_6_ is monoclinic (*C*12/*m*1), with average crystallite sizes of 49 and 77 nm, respectively. Structural studies have been additionally performed by Raman spectroscopy revealing optical phonons typical for vibrations of Te^6+^/W^6+^O_6_ octahedra. Both SNTO and SNWO possess high values of dielectric constants (341 and 308, respectively) with low dielectric loss (0.06 for SNWO) at a frequency of 1 kHz. These values decrease exponentially with the increase of frequency to 1000 kHz, with the dielectric constant being around 260 for both compounds and dielectric loss being 0.01 for SNWO and 0.04 for SNTO. The Nyquist plot for both samples confirms the non-Debye type of relaxation behavior and the dominance of shorter-range movement of charge carriers. Magnetic studies of both compounds revealed antiferromagnetic behavior, with Néel temperature (T_N_) being 57 K for SNWO and 35 K for SNTO.

## 1. Introduction

Complex metal oxides have a great fundamental and practical interest. They attract a great attention of the researchers due to the strong correlation between chemical composition, features of the crystal structure, magnetic, electrical and functional properties [1,2,3]. Nanostructured magnetic compounds have been investigated thoroughly as they can possess different physical and chemical properties compared to their bulk counterparts [4,5]. For example, many research groups [6,7,8,9,10,11,12] have shown that the transition temperature in antiferromagnetic materials (T_N_—Néel temperature) increases with the reduction of particle size. Sometimes, in the case of antiferromagnetic materials, additional transition might be observed [13] or spin canting effect might take place [14,15]. This behavior is usually not reported in bulk forms. Alterations can additionally be observed in dielectric properties and usually they are explained by changes in size, shape and particle or grain boundaries [16,17,18]. Concerning dielectric properties, it is desired to produce a material with high value of dielectric constant (permittivity) and low value of dielectric loss [19,20,21,22]. High-κ dielectric materials are materials with high value of dielectric permittivity. They are used in semiconductor manufacturing processes where they replace widely-used silicon dioxide as gate dielectrics. This allows the increase of the gate performance in term of capacitance [23]. However, most of these materials are manufactured as composites with polymers, such as PVA or PVDF, to increase the value of dielectric constant and decrease the value of dielectric loss [24]. High-κ dielectric polymer composites have been reported to be successful in various applications [25,26,27,28,29,30].

The other thing that can be tailored by particle size control is a value of optical band gap. This has shown a similar trend as transition temperature: It increases with a decrease in particle size [31]. Double perovskite materials are usually reported as semiconductors, with band gap values from 3 to 6 eV [32].

All of the above mentioned properties arise from specific crystal structure and are strongly correlated to it. Perovskite structure is summarized by general formula ABX_3_, where A is large cation such as alkali or earth alkali, B is small cation mostly transition metal and X stands for oxide, sulfide or halide anion [33]. Multiple substitutions could be performed at either A, B or X sites. In A_2_B’B’’O_6_ double perovskites, half of the B site is occupied by one transition metal cation, such as Ni^2+^ and the other half is occupied by some other transition metal cation, such as W^6+^ or a semimetal like Te^6+^ [34]. Here, Ni^2+^ possess a finite magnetic moment arising from unpaired d electrons, while Te^6+^ and W^6+^ are non-magnetic since their d orbitals are empty. This combination might produce some novel effects in single material, which is why these materials have been of a great interest during the last couple of decades.

Sr_2_NiWO_6_ (SNWO) and Sr_2_NiTeO_6_ (SNTO) have been studied since the 1960s and 1970s of the twentieth century and mostly were prepared in the bulk polycrystalline form or in the form of single crystals. SNWO was previously prepared only once in 2016 in nanocrystalline form using the sol-gel method by Xu et al. [35] and its photocatalytic activity was studied. It was first prepared by Fresia et al. [36] in 1959, then it was studied in the 1960s by Brixner [37], and Nomura [38,39], in the 1970s by Köhl [40] and later by Todate [41], Iwanaga [42], Gateshki [43], Tian [44] and more recently by Liu [45], Blum [46] and Rezaei [47]. SNTO was among first studied by Köhl [48], Lentz [49], Rossman [50] and later studied by Todate [41], Iwanaga [42], Ortega San-Martin [51] and Orayech [52].

Mostly the structure and magnetic properties have been investigated for these compounds with lack of knowledge in the dielectric part. Therefore, in this contribution our goal is to clarify dielectric properties and to investigate the effects that occur due to size reduction to nanoscale. For the first time we give a detailed explanation of dielectric properties for both compounds and show effects of size reduction on the magnetic properties.

## 2. Results and Discussion

### 2.1. Powder X-ray Diffraction

**Figure 1:**
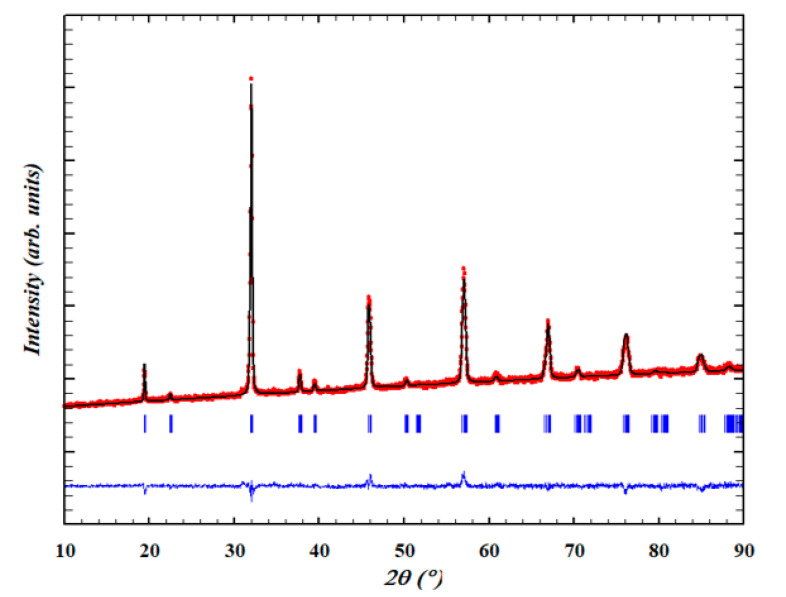
Calculated (black) vs. experimental (red) powder X-ray diffraction pattern for Sr_2_NiTeO_6_.

**Figure 2:**
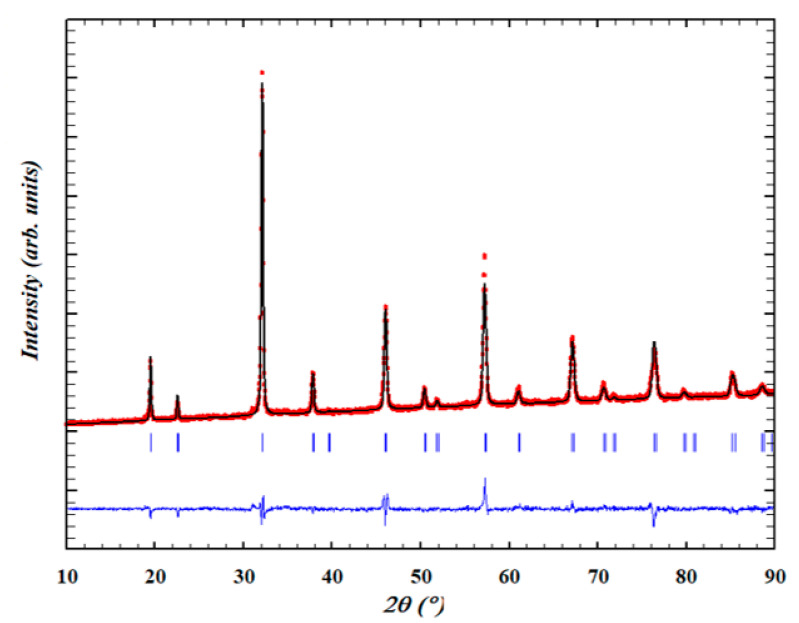
Calculated (black) vs. experimental (red) powder X-ray diffraction pattern for Sr_2_NiWO_6_.

Powder X-ray diffraction has been performed in order to investigate phase purity, crystal structure as well as microstructural parameters of as synthesized materials. The Rietveld refinement was conducted along with microstructural analysis [53], and the typical Rietveld output plots are given in Figure 1 and Figure 2 whilst the results of the refinement are summarized in Table 1, Table 2, Table 3 and Table 4. Both figures reveal phase purity of as synthesized double perovskites since all observed Bragg reflections were correctly described by the calculated curve based on the assumed structural models. Only in Figure 2 are several spikes in the difference curve observed; however, they are most probably attributed to some small preferred orientation.

Sr_2_NiWO_6_ crystallized in tetragonal centrosymmetric space group *I*4/m with lattice parameters equal to a = 5.5644(2), c = 7.9025(4) Å, while Sr_2_NiTeO_6_ crystallized in monoclinic centrosymmetric space group *C*12/*m*1 with lattice parameters: a = 9.663(1), b = 5.6132(2), c = 5.5833(2) Å, β = 125.32(1)°. Although investigated compounds differ significantly in terms of crystal symmetry, their unit cell volume values are quite close.

Comparison of atomic positions extracted by Iwanaga et al. [42] for bulk SNTO with this work, presented in Table 2, reveals quite different values, although they correspond to the same space group. As opposed to nanocrystalline SNTO reported in Table 2, in bulk as reported in [42] there are two different positions for magnetically-active Ni^2+^ cation (2a and 2d), which could result in different magnetic properties.

**Figure 3:**
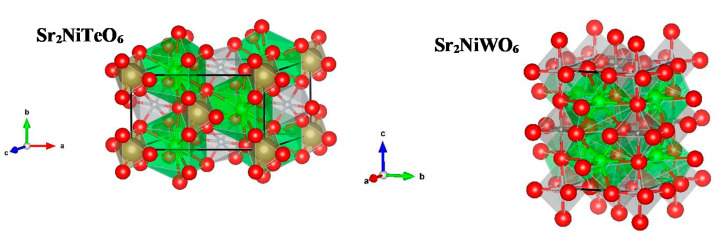
Crystal structures of the synthesized compounds visualized by VESTA [49].

Figure 3 shows the crystal structures of as synthesized compounds visualized by VESTA software [54] and Table 4 shows selected interatomic distances calculated by the Rietveld method. SNTO shows structural arrangement consisting of layers of TeO_6_ octahedra centered in corners of bc planes, mutually linked with NiO_6_ octahedron, centered in the middle of bc rectangle. The 3D structure was further composed by alternate linking of tilted TeO_6_-NiO_6_ octahedra along the [100] direction. The voids formed with such stacking were filled by SrO_12_ cuboctahedron. SNWO is rock-salt ordered with the arrangements of a mutually slightly-rotated corner-sharing NiO_6_-WO_6_ octahedra along the c-axis due to the tetragonal distortion.

In monoclinic Sr_2_NiTeO_6_ there are two types of octahedra that differ according to their lengths: shorter (smaller volume) TeO_6_ octahedra and slightly larger NiO_6_ octahedra. Moreover, both octahedra are highly distorted since the apical line is not perpendicular to the equatorial plane. Tetragonal Sr_2_NiWO_6_ comprises NiO_6_ octahedra and WO_6_ octahedra that differ to each other according to their bond lengths: Equatorial bond lengths in NiO_6_ are visibly shorter compared to WO_6_, while the apical lengths of NiO_6_ are slightly longer than in WO_6_, as seen from the Table 4. It is noteworthy that SrO_12_ cuboctahedron is more distorted (more different Sn-O bond lengths) for SNTO in comparison with SNWO which is the result of lower symmetry of SNTO. The line broadening analysis performed within the Rietveld refinement reveals that SNTO shows higher crystallinity (average crystallite size equals to 77 nm) compared to SNWO (49 nm). Although the crystallinity is higher for SNTO, its microstrain level is also higher compared to SNWO, which is an unexpected and interesting finding.

### 2.2. Unpolarized Raman Spectroscopy

**Figure 4:**
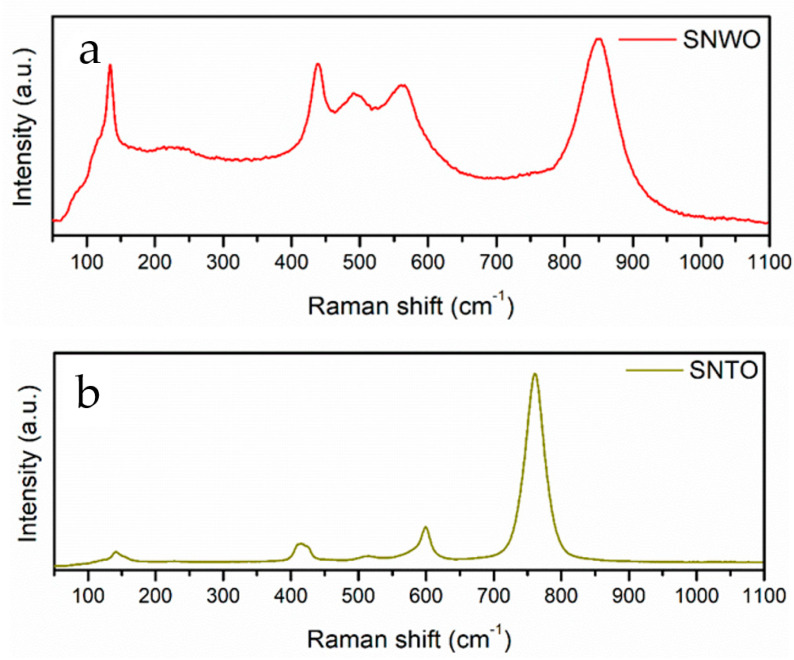
Raman spectra of the Sr_2_NiWO_6_ (SNWO, (**a**)) and the Sr_2_NiTeO_6_ (SNTO, (**b**)).

Raman spectroscopy was conducted in order to inspect bond length stretching and possible phonon confinement effects that frequently occur in nanocrystalline materials. Figure 4 shows Raman spectra of double perovskites: tetragonal Sr_2_NiWO_6_ (SNWO in Figure 4a), and monoclinic Sr_2_NiTeO_6_ (SNTO in Figure 4b). According to Kroumova et al. [55] and based on the space group, Sr_2_NiTeO_6_ has 7A_g_ + 5B_g_, 12 first-order Raman active modes (optical modes) in total. Sr_2_NiWO_6_ has 3A_g_ + 3B_g_ + 3^1^E_g_ + 3^2^E_g_, which is also 12 first-order Raman active modes in total. Only four bands out of 12 for both SNWO and SNTO appear in Figure 4. Peaks appearing in the range from 100–150 cm^−1^ are assigned to lattice translational modes [51]. They are observed at 134 cm^−1^ for SNWO and at 142 cm^−1^ for SNTO. Peaks in the range 350–450 cm^−1^ are assigned to ν_5_ mode, which appears due to oxygen bending in octahedra [56,57]. It is observed at 440 cm^−1^ for SNWO and at 416 cm^−1^ for SNTO. Since the Te^6+^-O bond is the shortest bond for the studied SNTO, it is also the strongest bond for this compound and higher frequencies in the spectrum should be primarily assigned to ν_1_ and ν_2_ vibrations of the Te^6+^O_6_ octahedron. Hence, for SNTO, the frequencies corresponding to vibrations of Ni^2+^O_6_ octahedron are not expected and indeed were not observed. Peaks assigned to ν_2_ mode are due to asymmetric stretching that appear in the range 470–650 cm^−1^ according to Silva et al. [56]. This mode is represented by two peaks for both compounds, at 497 and 564 cm^−1^ for SNWO and at 510 and 600 cm^−1^ for SNTO. The highest wavenumber appearing within spectra (850 cm^−1^ for SNWO and 762 cm^−1^ for SNTO) is assigned to ν_1_, symmetric stretching of the WO_6_ and TeO_6_ octahedra, respectively. Optical phonons for both compounds are summarized in Table S1 in the Supporting file. Similar to Silva et al. [56], we also observe a larger difference in the ν_1_ vibration between Te^6+^- (*d*^10^) and W^6+^-based (*d*^0^) compounds. These authors correlated the increase of wavenumber in W-containing compounds compared to Te-containing compounds with the increase of the force constant (bonding energy) of the W-O bond in the WO_6_ octahedron. The Te^6+^ cation has a fully-occupied *d*^10^ orbital configuration, which avoids the formation of π-type Te-O bonds. On the other hand, the W^6+^ cation has a *d*^0^ orbital configuration, allowing the overlap of the t_2g_ orbitals The increase of force constant/bonding energy occurs due to the overlapping of t_2g_ orbitals of the octahedrally-coordinated W^6+^ cation [56]. Similar arguments are valid for the absence of vibrations of Ni^2+^O_6_ octahedron for SNWO. Although, the shortest bond in this compound is the equatorial Ni-O bond (1.873(2) Å), the formation of π-type W-O bonds occurred, which resulted in the increase of W-O bonding energy, thus Raman modes of W^6+^O_6_ dominate. Ayala et al. [58] studied tetragonal and monoclinic double perovskites and came to conclusion that these spectra resemble to those of cubic *Fm*-3*m* perovskites. The similarity comes from the fact that tetragonal and monoclinic structures arise from the small distortions of the cubic cell [56]. When comparing our SNWO spectrum in Figure 7a with the bulk SNWO reported by Manoun et al. [59], there is a progressive shift to lower wavenumbers (lower energies); line shapes are broadened and asymmetric on the lower energy side. Additionally, symmetry breaking becomes more emphasized. These effects occur due to the reduction of size and quantum confinement effect [60,61,62]. Actually, the impact of the size reduction on the Raman spectra is quite visible, since SNWO contains smaller crystals compared to SNTO and thus its Raman bands are also broader, reflecting a higher disorder in SNWO.

### 2.3. Scanning Electron Microscopy and Energy Dispersive X-ray Spectroscopy

**Figure 5:**
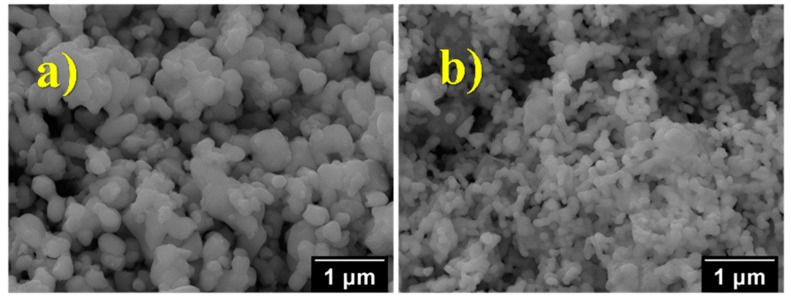
SEM images of SNTO (**a**) and SNWO (**b**).

To investigate the morphology and chemical composition of as-prepared compounds, scanning electron microscopy (SEM) and energy dispersive X-Ray spectroscopy (EDX) measurements were performed. SEM images are shown in Figure 5, EDX spectra for both compounds are shown in Figure S1 and Figure S2 in SI.

SEM images show irregularly round-shaped particle agglomerates for both SNTO and SNWO at the same magnification (1 μm). It is clearly visible that SNTO particles are larger than SNWO particles, which is in accordance with microstructural analysis conducted using the Rietveld refinement method. The surface morphology of the particles represents the random distribution of the various size of the grains and visible grain boundaries.

The energy dispersive X-ray spectrum and elemental mapping suggest the presence of all elements is similar to the base composition confirming that both synthesized compounds SNTO and SNWO are free from impurity. Figure S1 shows that Sr_2_NiWO_6_ consists of 61.2 at % O, 19.7 at % Sr, 10.8 at % Ni and 8.3 at % W, which nearly corresponds to the proposed chemical formula. It is similar with Sr_2_NiTeO_6_, which consists of 63.4 at % O, 18.1 at % Sr, 9.9 at % Ni and 8.6 at % Te (Figure S2).

### 2.4. Dielectric Properties

**Figure 6:**
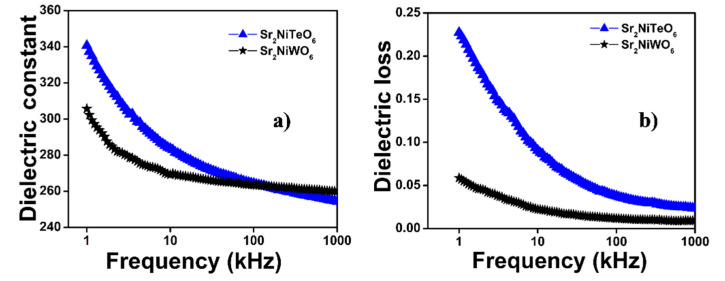
(**a**) Dielectric constant and (**b**) dielectric loss of Sr_2_NiWO_6_ and Sr_2_NiTeO_6_ ceramics at room temperature.

Figure 6a displays dielectric constant and Figure 6b dielectric loss versus frequency for SNTO and SNWO ceramic samples at room temperature. It is observed that the dielectric constant (ε_r_) and dielectric loss (tan δ) are high at lower frequency regime and decreases at higher frequency regime, indicating that the familiar dielectric dispersion phenomenon as observed in the case of normal ferroelectrics. Frequency-temperature dependence of ε_r_ and tan δ is associated with various polarization effects, such as ionic, dipolar, electronic and space charge that appears at multiple levels of material reaction due to short and long-range movement of mobile charges. At low-frequency regions, the space charge and dipolar polarization are at the peak and interfacial polarizations efficiently add to the upper value of ε_r_. In the high-frequency region, the electronic polarizations become most significant compared to other polarizations, leading to invariant dielectric constant. The decrease in the value of dielectric constant with the rise in frequency may be due to the dipoles unable to follow the rapid oscillating field [63]. The dielectric loss has a similar type of trend as ε_r_ in the low-frequency regime, where the loss is high due to the influence of compositional disorder, leading to a relaxation phenomenon [64]. Mutual comparison between SNTO and SNWO reveals that SNWO has a lower value of ε_r_ (308 compared to 341 at 1 kHz), while its dielectric loss is significantly lower (0.06 compared to 0.23). Moreover, frequency-dependent quantities show milder decrease for SNWO, indicating its better perspective for electronic applications.

### 2.5. Impedance Spectroscopy

**Figure 7:**
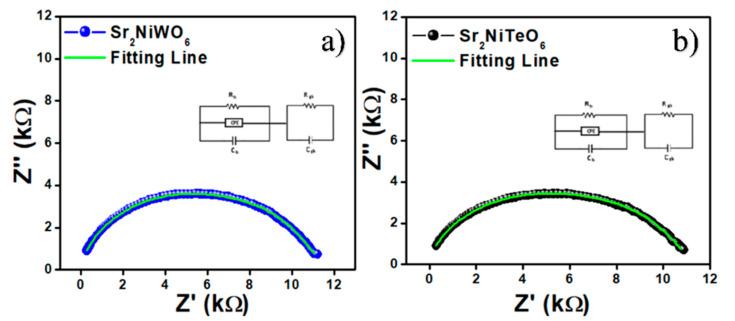
Nyquist plot of (**a**) Sr_2_NiWO_6_ and (**b**) Sr_2_NiTeO_6_ ceramics at room temperature. In the inset, the equivalent electrical circuits are displayed.

**Figure 8:**
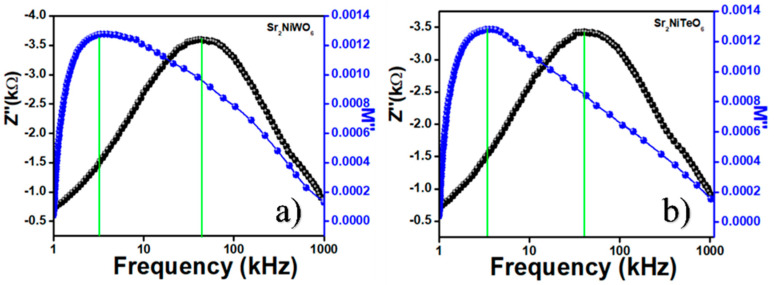
Frequency dependent imaginary part of impedance and modulus: (**a**) Sr_2_NiWO_6_ and (**b**) Sr_2_NiTeO_6_ ceramics.

The Nyquist plot in general has several semicircular arcs, whereby the first circle (higher frequency) represents the grain/bulk effect, the second circle (intermediate frequency) represents the grain boundary effect and the third circle (lower frequency) represents the electrode effect. Normally, depressed semicircular arcs are obtained with the center below the abscissa implying departure from Debye type behavior. There are several factors, such as atomic defect distribution, stress–strain phenomena, grain boundary, and grain orientation, which can be correlated to the above-described non-ideal behavior. The depressed semicircles also provide evidence of polarization phenomena with the allocation of relaxation times. In Debye-type behavior, the center of the circle lies exactly on the real *Z*-axis [65]. Figure 7 shows the depressed semicircle and the non-Debye type of relaxation phenomena are occurring for both synthesized materials. The commercial Z Smipwin software is used to fit the experimental impedance data to an equivalent circuit model bearing a constant phase element (CPE). The parameters derived from fitting are grain resistance, grain capacitance, grain boundary resistance, and grain boundary capacitance and corresponding values are listed in Table 5. Figure 8 shows the frequency-dependent M′′ and Z′′ plot of (a) SNTO and (b) SNWO ceramics. It represents the effect of longer- and shorter-range movement of charge carriers on the relaxation process. The shorter-range charge carrier dominancy is interpreted by the M′′ and Z′′ peaks mismatch, whereas if the M′′ and Z′′ peaks exactly match then it is related to the long-range motion of charge carriers [66]. In Figure 8a,b, it is depicted that the peaks of the M′′ and Z′′ of SNWO and SNTO coincides in the same position, suggesting the dominance of shorter-range movement of charge carriers.

### 2.6. Magnetic Properties

**Figure 9:**
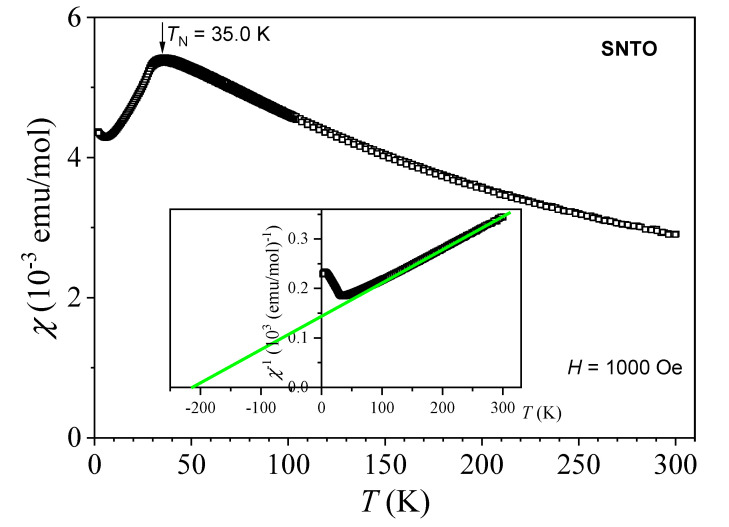
Temperature-dependent susceptibility and inverse susceptibility (inset) of SNTO.

Temperature-dependent susceptibility of SNTO measured in a magnetic field of 1000 Oe is shown in Figure 9. The susceptibility increases as temperature decreases from room temperature down to 35 K. At Néel temperature (T_N_) = 35.0 K, the susceptibility exhibits a maximum, in agreement with already reported antiferromagnetic ground state of SNTO [42]. No difference between zero-field-cooled (ZFC) and field-cooled (FC) susceptibility was observed. The measured susceptibility in high temperature region (T > 100 K) was analyzed using a Curie-Weiss law χ = C/(T−ϑ). The result, presented as a full line in χ^−1^ vs. T plot (inset in Figure 9), gave the Curie constant C = 1.4 emu K/mol and Curie–Weiss temperature ϑ = −210 K. The effective magnetic moment $\mu \;=\;\sqrt{8\;C}=3.3\mu B$ in agreement with the expected value for Ni(II) ions with non-zero orbital contribution L [67]. The magnetization curve M(H) at 2 K (Figure S3) is linear up to the maximal magnetic field of 50 kOe with very small magnetization (0.04 μB at 50 kOe), as expected for the antiferromagnetic ground state.

**Figure 10:**
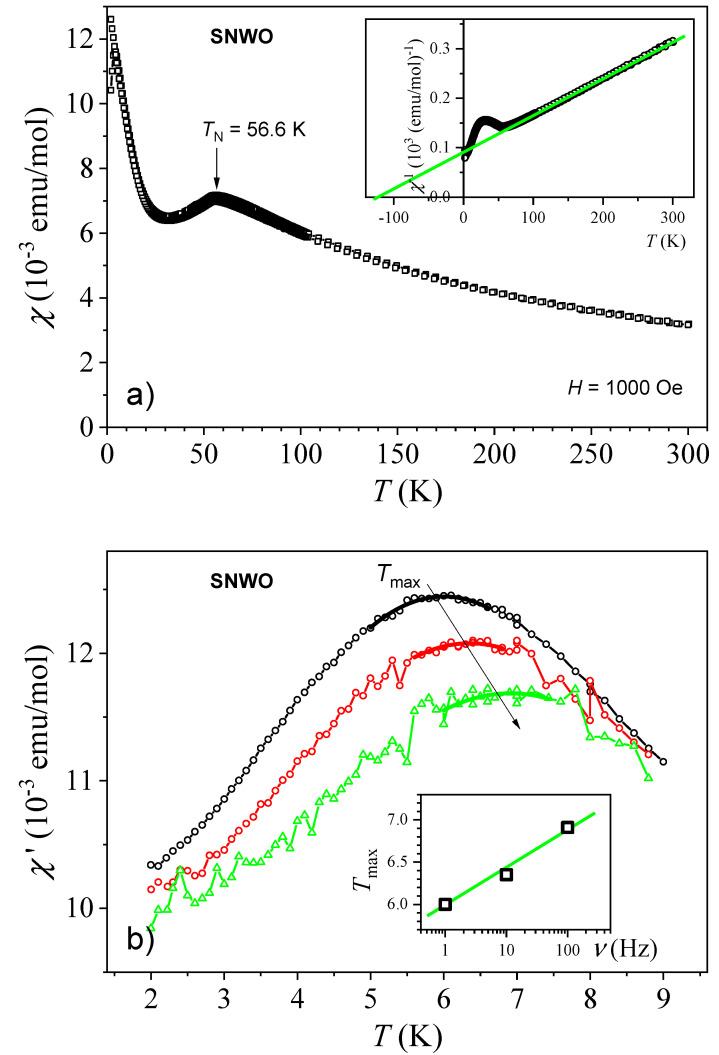
Temperature-dependent susceptibility and inverse susceptibility (inset) of SNWO (**a**). AC susceptibility (susceptibility in alternating magnetic field) around the low temperature peak (**b**) and shift of the maximum of AC susceptibility with frequency (inset in (**b**)).

In Figure 10a the temperature-dependent magnetic susceptibility is shown for SNWO. A local maximum of susceptibility (Néel temperature) appears here at a higher Néel temperature (T_N_) = 56.6 K. The magnetization M(H) curve at 2 K is linear as in SNTO (Figure S3), with a very small value of the magnetization in a maximal field of 50 kOe. Both results speak in favor of antiferromagnetic ground state in SNWO, as already reported in the literature [42,46]. A slightly higher Néel temperature (56.6 K) than the literature data for bulk material (53 K in [42], 54 K in [46]) might be ascribed to a nanosized material in our case. Various researchers have already described that Néel temperature increases with the decrease in crystallite size [6,7,8,9,10,11,12].

Above T_N_, the susceptibility follows the Curie–Weiss law (inset in Figure 10). The same analyses as performed in the case of SNTO (Curie-Weiss fit), which gave the Curie–Weiss temperature ϑ = −125 K and effective magnetic moment of 3.2 μB. These values are similar to the literature data [42,46].

However, below T_N_, the susceptibility starts to increase once again. The ZFC/FC splitting can be observed below 6 K, which was, to our knowledge, not reported yet in literature. In order to investigate this low temperature signal in more detail, we performed additional AC susceptibility measurements (susceptibility in alternating magnetic field) shown in Figure 10b. The AC susceptibility has been measured at three different frequencies of the applied magnetic field—1, 10, and 100 Hz. The amplitude of AC magnetic field was 6 Oe. The maximum of AC susceptibility is at approximately T_max_ = 6 K for the frequency of the applied field of 1 Hz, and shifts to higher temperatures with frequency. This feature is typical for a frustrated magnetic system, where both ferromagnetic and antiferromagnetic interaction are present in the system simultaneously, and (or) there is some degree of disorder in a distribution of magnetic moments. [68] The relative shift of T_max_ with frequency (inset in Figure 10b), $Г\;=\;\frac{\Delta T_{\max}}{T_{\max\left(1\;\mathrm{Hz}\right)}\;\Delta \log_{v}}=0.07$, falls somewhere between the values of typical spin glasses and superparamagnetic systems [69]. A very similar signal can be found also in NiO nanoparticles [68]. We tentatively ascribe this low temperature signal to the magnetism of uncompensated nickel ions on the surface of nanoparticles, where the perfect antiferromagnetically-ordered environment around the magnetic ions, as established in bulk compounds, is corrupted.

## 3. Materials and Methods

Materials. The following commercially available chemicals were used without further purification: Ammonium tungsten oxide hydrate and ammonium tellurate from Alfa Aesar, Germany; strontium(II) nitrate, and nickel(II) nitrate hexahydrate from Sigma Aldrich, Germany; citric acid monohydrate 99.9% from T.T.T., Croatia; and concentrated ammonia solution 25% from Gram-Mol, Croatia.

**Scheme 1:**
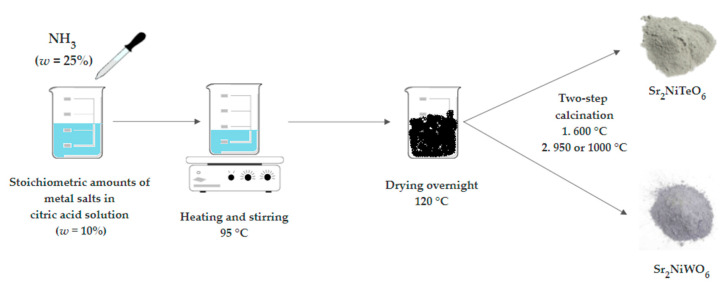
Schematic representation of sol-gel synthesis of double perovskites Sr_2_NiTeO_6_ and Sr_2_NiWO_6_.

Synthesis. Double perovskites were synthesized using aqueous sol-gel citrate method, previously reported for triple perovskites [57,70]. Stoichiometric amounts were dissolved in 10% citric acid solution (10 g of citric acid in 100 mL of MiliQ water) for synthesis of SNTO: A total of 2 mmol of strontium(II) nitrate, 1 mmol of nickel(II) nitrate hexahydrate and 1 mmol of ammonium tellurate. For synthesis of SNWO, instead of ammonium tellurate, 1/12 mmol of ammonium tungsten oxide (molecular formula: H_40_N_10_O_41_W_12_•xH_2_O) was used. The value of pH was adjusted to 5 using concentrated ammonia solution (pH-meter 211, HANNA, Zagreb, Croatia). Reaction mixture was then evaporated at 95 °C, with constant stirring on a magnetic stirrer (IKA RCT Basic, Staufen, Germany), until black resin was formed. Black resin was further dried in a drying oven (Instrumentaria ST-05, Sesvete, Croatia) at 120 °C for 24 h and grinded in a mortar with a pestle. Obtained black powder was first calcined at 600 °C for 8 h and then at 950 °C (SNTO) or 1000 °C (SNWO) for 12 h (Furnace SN 342689, Nabertherm GmbH, Lilienthal, Germany). Heating rate for both calcination steps was 2 °Cper minute. Synthesis procedure is represented in Scheme 1 below.

Preparation of pellets for dielectric measurements. The calcined powder was converted into fine particles and 5 wt % of binder polyvinyl alcohol (PVA) was mixed. Further, disk-shape pellets comprising a diameter of 10 mm and a thickness of 1.2 mm were sieved employing a hydraulic press (Model number: 220-93614-01, Shimadzu, Kyoto, Japan) with cold pressure of 4.0 × 10^6^ Nm^−2^. Subsequently, the green pellets were sintered at 1250 °C for 4 h at a heating rate of 8 °C/min in a tube furnace (Model number: SH-FU-50TS, SH Scientific, Daejeon, Korea). One of the pellets of each composition was taken and rubbed with zero emery paper to make the opposite sides smooth and parallel. The high-quality silver paint was painted on both opposite sides of each pellet that served as an electrode and further electrical characterization was performed.

Methods. Powder X-Ray diffraction patterns were collected on a Panalytical XPert Pro Diffractometer with *θ*–2*θ* geometry (Malvern Panalytical, Malvern, UK), using monochromatized CuKα radiation (40 kV, 40 mA) at 292(2) K, in the range of 2*θ* from 10°–90° with the step size of 0.02.

Unpolarized Raman spectroscopy was performed at room temperature in backscattering geometry using a Renishaw inVia Raman microscope system (Reinshaw GmbH, Pliezhausen, Germany) with a HeNe laser (633 nm) for excitation, in the range 50–1100 cm^−1^ with 2 mW laser power. Samples were placed on the microscope slides in a powder form.

The surface morphology was investigated using a scanning electron microscope (M/S TESCAN Mira 3, Brno, Czech Republic).

The resistive and capacitive parameters were fetched from a computer-coupled phase-sensitive meter (Hioki IM3570, Nagano, Japan). The electrical properties of the prepared pellets were examined over a frequency sweep (1 kHz–1 MHz) and at room temperature.

Magnetic properties were investigated using a MPMS-XL-5 magnetometer (Quantum Design, San Diego, CA, USA). All presented data were corrected for diamagnetic contribution estimated from Pascal’s constants [71].

## 4. Conclusions

This study was performed to clarify the impact of size reduction on magnetic properties and to investigate dielectric properties of synthesized Ni-based double perovskites. Most of the reported literature deal with bulk materials and their magnetic properties along with detailed crystal structure analysis. Since miniaturizing and enhancing the performance has become a trend in the production of electronic devices, our motivation was to decrease crystallite size of already known compounds and to shed some light on their magnetic and electrical properties.

Double perovskites Sr_2_NiTeO_6_ and Sr_2_NiWO_6_ both possess magnetically active Ni^2+^ cation and magnetically inactive d^10^ Te^6+^ (diameter of 0.56 Å for coordination number 6 [72]) and d^0^ W^6+^ (diameter of 0.60 Å for coordination number 6 [72]) cations, respectively. Coexistence of these two species in a single-phase material is one of the conditions for coupling of magnetic and dielectric properties within the same material, which leads to multifunctionality.

So far, we have successfully synthesized, using modified sol-gel route phase pure Ni-based perovskites, Sr_2_NiTeO_6_ and Sr_2_NiWO_6_. Since the ionic radii values of octahedrally-coordinated Te^6+^ and W^6+^ are similar [72], it would be expected that their crystal structures are also similar. The value of their Goldschmidt tolerance factors are the same up to fourth decimal digit (*t* = 0.9889) [33]. However, even the small difference in ionic radii can produce different structures. Thus, Sr_2_NiWO_6_ is crystalized in the tetragonal crystal system (*I*4/*m*) with an average crystallite size of 49 nm, while Sr_2_NiTeO_6_ is monoclinic (*C*12/*m*1) with 77 nm the average crystallite size. On the other hand, monoclinic and tetragonal structures are both formed because of small distortions of the cubic cell, so their similarity is still preserved.

Both compounds are antiferromagnetically ordered, as it has already been reported before for their bulk and single crystal forms. Sr_2_NiTeO_6_ behaves the same as nanocrystalline and bulk material, having the same value of Néel temperature (T_N_ = 35 K). However, Sr_2_NiWO_6_ possesses a slightly larger value of T_N_ (56.6) than single crystal (53 K) and bulk (54 K) forms, which often occurs due to size reduction.

For the first time we reported the results of dielectric measurements for these compounds. Both compounds have high values of dielectric constants (341 for Sr_2_NiTeO_6_ and 308 for Sr_2_NiWO_6_) at room temperature and low frequency (1 kHz), which classifies them as high-κ dielectrics. Moreover, the W-containing compound is promising for application in electronic devices, since its dielectric loss of 0.06 at 1 kHz is significantly lower compared to its Te counterpart. Impedance spectroscopy results point out that the non-Debye type of relaxation phenomena are occurring for both compounds, suggesting also the dominance of the shorter-range movement of charge carriers.

These materials could be promising candidates to use in electronic devices because they possess both dielectric and antiferromagnetic behavior. Additionally, the size reduction to nanoscale favors the production of smaller devices with enhanced functionality.

## Figures and Tables

**Table 1:** Crystallographic data and Rietveld refinement parameters obtained from the XRD patterns of the synthesized compounds.

Chemical Formula	Sr_2_NiTeO_6_	Sr_2_NiWO_6_
Space group	*C*12/*m*1 (12)	*I*4/*m* (87)
Molecular weight	457.53	513.77
Z	2	
Crystal system	Monoclinic	Tetragonal
Lattice parameters (Å)	a = 9.663(1) b = 5.6132(2) c = 5.5833(2) β = 125.32(1)°	a = 5.5644(2) c = 7.9025(4)
Cell volume (Å^3^)	247.09(4)	244.68(2)
Calculated density (g/cm^3^)	6.15	6.97
Data collection range	10–90°	
No. of parameters refined	24	18
No. of bond lengths restrained	11	0
No. of bond angles restrained	0	0
Average apparent crystallite size (nm)	77	49
Average apparent microstrains (×10^−4^)	17.462	11.828
Phase composition (wt %)	100	
R_B_ (%)	5.84	6.1
Conventional R_p_, R_wp_, R_e_ (%)	27.0, 16.5, 11.8	19.9, 14.4, 9.31
*χ* ^2^	1.961	2.398

**Table 2:** Structural parameters for Sr_2_NiTeO_6_ extracted at room temperature (292 K). The site occupancies are expressed in terms of the ratio m:M—site multiplicity:multiplicity of a general position (8).

Atom	Wyckoff Position	x ^a^	y ^a^	z ^a^	B (Å^2^) ^b^	Occupancy
**Sr**	4i	0.751(1)	0	0.235(2)	0.9(1)	12
**Ni**	2d	0	1/2	1/2	0.1(1)	14
**Te**	2a	0	0	0	1.8(1)	14
**O1**	8j	0.0264(9)	0.2443(9)	0.266(1)	1.7(2)	1
**O2**	4i	0.2436(7)	0	0.301(2)	1.7(2)	12

^a^ Atomic coordinates in 3D space; ^b^ Debye-Waller factor.

**Table 3:** Structural parameters for Sr_2_NiWO_6_ extracted at room temperature (292 K). The site occupancies are expressed in terms of the ratio m:M—site multiplicity:multiplicity of a general position (16).

Atom	Wyckoff Position	x ^a^	y ^a^	z ^a^	B (Å^2^) ^b^	Occupancy
Sr	4d	0	1/2	1/4	1.30(7)	14
Ni	2a	0	0	0	0.9(1)	18
W	2b	0	0	1/2	1.19(5)	18
O1	8h	0.269(3)	0.200(3)	0	2.35(4)	12
O2	4e	0	0	0.255(2)	2.35(4)	14

^a^ Atomic coordinates in 3D space; ^b^ Debye-Waller factor.

**Table 4:** Selected interatomic distances for synthesized compounds.

Compound	Bond Type	Bond Length (Å)
**Sr_2_NiTeO_6_**	Sr-O1 x2	2.707(7)
	Sr-O1 x2	2.689(1)
	Sr-O1 x2	2.909(1)
	Sr-O1 x2	2.900(8)
	Sr-O2 x1	2.558(2)
	Sr-O2 x1	3.026(1)
	Sr-O2 x2	2.837(3)
	Ni-O1 x4	2.052(7)
	Ni-O2 x2	2.047(6)
	Te-O1 x4	1.929(6)
	Te-O2 x2	1.947(5)
**Sr_2_NiWO_6_**	Sr-O1 x4	2.606(1)
	Sr-O1 x4	2.988(2)
	Sr-O2 x4	2.7825(1)
	Ni-O1 x4	1.873(2)
	Ni-O2 x2	2.0151(1)
	W-O1 x4	2.099(2)
	W-O2 x2	1.9361(1)

**Table 5:** The values of grain resistance and grain capacitance from the Nyquist plot fitting.

Composition	R_g_ (Ω) ^a^	C_g_ (F) ^b^	R_gb_ (Ω) ^c^	C_gb_ (F) ^d^
Sr_2_NiWO_6_	1.054 × 10^4^	1.270 × 10^−10^	1102	1.083 × 10^−8^
Sr_2_NiTeO_6_	1.016 × 10^4^	1.268 × 10^−10^	1179	1.057 × 10^−8^

^a^ Grain resistance; ^b^ Grain capacitance; ^c^ Grain boundary resistance; ^d^ Grain boundary capacitance.

## Supplementary Materials

The following are available online, Table S1: Optical phonons (in cm^−1^) of phase pure SNWO and SNTO, Figure S1: EDX spectrum of SNWO, Figure S2: EDX spectrum of SNTO, Figure S3: Magnetization curves of SNTO and SNWO at 2 K.
