# Supplementary material for: Nanocrystalline Antiferromagnetic High-κ Dielectric Sr2NiMO6 (M = Te, W) with Double Perovskite Structure Type
Source: Molecules. 2020 Sep 2;25(17):3996. doi: 10.3390/molecules25173996 (PMC7504737; doi:10.3390/molecules25173996)
Supplement: Supplementary file 1 [file molecules-25-03996-s001.pdf]

Supporting information file for

Nanocrystalline Antiferromagnetic High-κ Dielectric Sr_2NiMO_6 (M = Te, W) with Double Perovskite Structure Type

Jelena Bijelić [1], Dalibor Tatar [1], Sugato Hajra [2], Manisha Sahu [2], Sang Jae Kim [2], Zvonko Jagličić [3,4] and Igor Djerdj [1,*]

[1] Department of Chemistry, Josip Juraj Strossmayer Univesity of Osijek, Cara Hadrijana 8/A, HR-31000 Osijek, Croatia; jelena.bijelic@kemija.unios.hr (J.B.); tatar.dalibor42@gmail.com (D.T.)
[2] Nanomaterials and System Lab, Major of Mechatronics Engineering, Faculty of Applied Energy Systems, Jeju National University, Jeju 63243, South Korea; sugatofl@outlook.com (S.H.); manishafl@outlook.com (M.S.); kimsangj@jejunu.ac.kr (S.J.K.)
[3] Institute of Mathematics, Physics and Mechanics, University of Ljubljana, Jadranska 19, SI-1000 Ljubljana, Slovenia; zvonko.jaglicic@imfm.si
[4] Faculty of Civil and Geodetic Engineering, University of Ljubljana, Jamova 2, SI-1000 Ljubljana, Slovenia
* Correspondence: igor.djerdj@kemija.unios.hr; Tel.: +385 31 399 975

Received: 07 August 2020; Accepted: 01 September 2020; Published: date

TableS1. Optical phonons (in cm^{-1}) of phase pure SNWO and SNTO.

Compound	Raman Shift (cm^{-1})	Assignment
**Sr_2NiWO_6**	134	Lattice (T-translational)
	440	ν_5
	497	ν_2
	564	
	850	ν_1
**Sr_2NiTeO_6**	141	Lattice (T-translational)
	416	ν_5
	510	ν_2
	600	
	760	ν_1

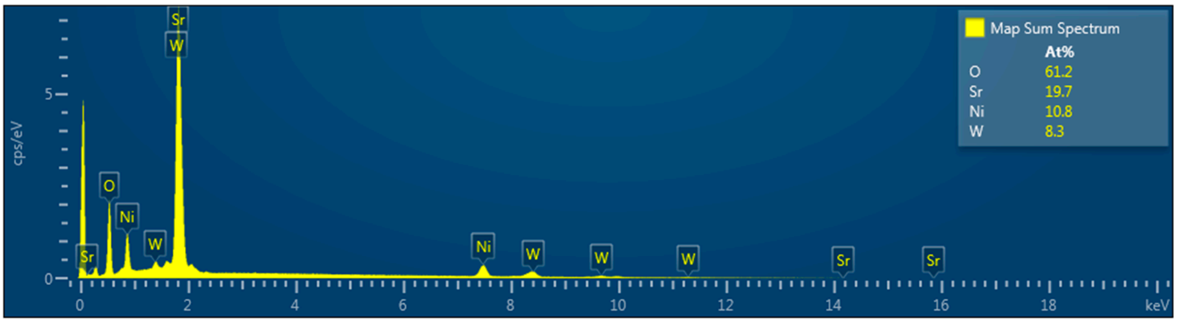

FigureS1. EDX spectrum of SNWO.

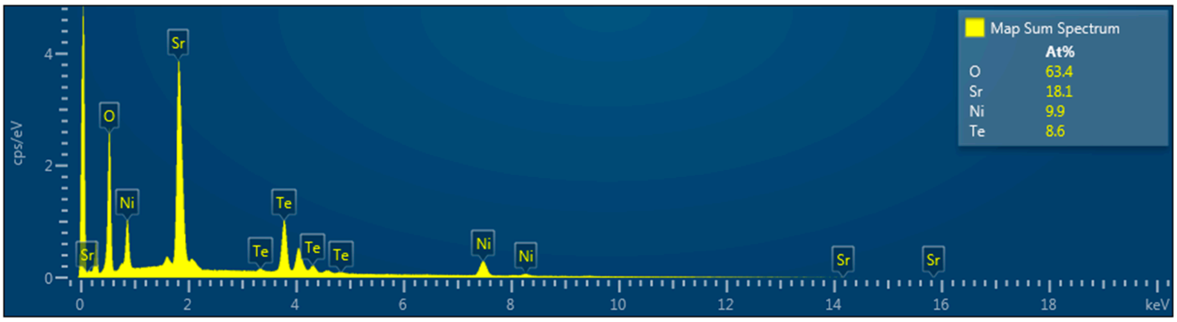

Figure S2. EDX spectrum of SNTO.

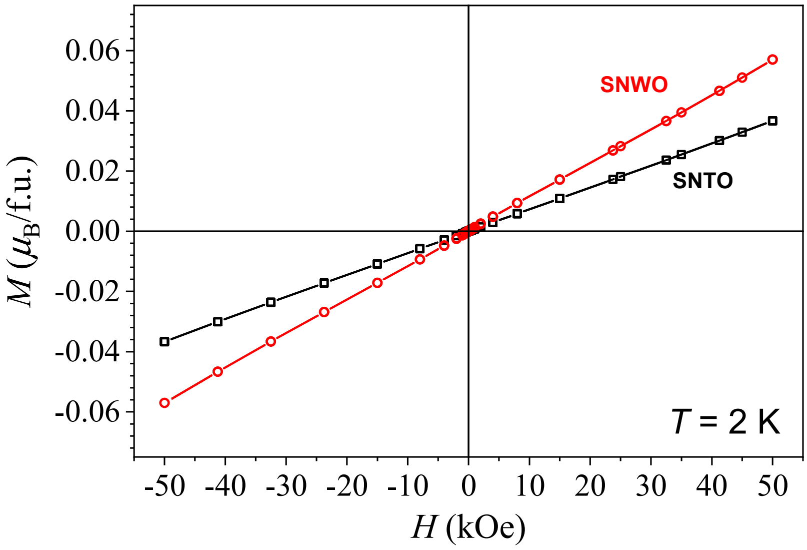

FigureS3. Magnetization curves of SNTO and SNWO at 2 K.
